# A High-Throughput Electrokinetic Micromixer via AC Field-Effect Nonlinear Electroosmosis Control in 3D Electrode Configurations

**DOI:** 10.3390/mi9090432

**Published:** 2018-08-26

**Authors:** Kai Du, Weiyu Liu, Yukun Ren, Tianyi Jiang, Jingni Song, Qian Wu, Ye Tao

**Affiliations:** 1 School of Electronics and Control Engineering, and School of Highway, Chang’an University, Middle-Section of Nan’er Huan Road, Xi’an 710064, China; dukai@chd.edu.cn (K.D.); liuweiyu@chd.edu.cn (W.L.); jn2012jenny@sina.com (J.S.); 2 State Key Laboratory of Robotics and System, Harbin Institute of Technology, West Da-zhi Street 92, Harbin 150001, China; jty_hit@sina.com (T.J.); tarahit@gmail.com (Y.T.); 3 Science and Technology on Reactor System Design Technology Laboratory, Nuclear Power Institute of China, Chengdu 610213, China; mercuryno.12@163.com

**Keywords:** electrokinetic micromixer, induced-charge electroosmosis, field-induced Debye screening, AC field-effect flow control, electrochemical ion relaxation

## Abstract

In this study, we make use of the AC field-effect flow control on induced-charge electroosmosis (ICEO), to develop an electrokinetic micromixer with 3D electrode layouts, greatly enhancing the device performance compared to its 2D counterpart of coplanar metal strips. A biased AC voltage wave applied to the central gate terminal, i.e., AC field-effect control, endows flow field-effect-transistor of ICEO the capability to produce arbitrary symmetry breaking in the transverse electrokinetic vortex flow pattern, which makes it fascinating for microfluidic mixing. Using the Debye-Huckel approximation, a mathematical model is established to test the feasibility of the new device design in stirring nanoparticle samples carried by co-flowing laminar streams. The effect of various experimental parameters on constructing a viable micromixer is investigated, and an integrated microdevice with a series of gate electrode bars disposed along the centerline of the channel bottom surface is proposed for realizing high-flux mixing. Our physical demonstration on field-effect nonlinear electroosmosis control in 3D electrode configurations provides useful guidelines for electroconvective manipulation of nanoscale objects in modern microfluidic systems.

## 1. Introduction 

Stirring two or more sample streams is crucial and challenging for clinical diagnostics, thermal management and drug development in micrometer scale [1]. A myriad of approaches have been discovered since the last decade to enhance sample mixing in microfluidic channels, including either active or passive micromixer, considering the driving mechanism upon which they work [2]. Because passive mixing stretches the two-phase contact surface, increases the time of interfacial mass exchange and perturbs the laminar streamlines between co-flowing buffer media by embedding solid obstacles into the microchannel, it is completely dependent on the molecular diffusion effect or chaotic convection [3]. On the contrary, active micromixers employ external energy input for arousing dynamic fluid motion to improve the mixing efficiency, such as Rayleigh streaming [4], magnetic [5], and electrokinetic [6,7,8].

Among them, electrohydrodynamic has been extensively exploited in microsystems to achieve flexible sample manipulation. In particular, recent progress of microelectronic processing makes the integration of compact microelectrode arrays into microchannels much easier, offering precious opportunities for exerting active Coulomb/dielectric forces on the fluid bulk during exposure to AC electric fields [9,10]. In contrast with dc electroosmotic (DCEO) slipping on insulating charged channel sidewalls [11,12,13], electroconvection emerges as a series of vortex flow along a discrete electrode array with voltage of merely several volts, so AC electrokinetics is able to achieve more flexible control on the local flow pattern, and is appropriate for some interesting applications, such as fluid transport [14,15,16], sample mixing [17,18,19] and generation of concentration-gradient [20] in microdevices. Taking into account the high degree of freedom controllability by adjusting the magnitude, phase relation and field frequency of the imposed voltage signal, both AC electrothermal (ACET) flow [21,22,23] and induced-charge electroosmosis (ICEO) [24,25,26] have been gaining unprecedented attention from the microfluidic community. 

The origin of ACET consists in smeared structural polarization [27,28,29]. Since electric properties are usually a function of local temperature elevation, Joule heating gives rise to inhomogeneous liquid conductivity and permittivity in applied AC fields. Both free and bound charges are induced by the interaction of the dielectric gradient with the AC fields, which also accounts for the dielectrophoretic force acting on each liquid element, so that ACET is a nonlinear electrokinetic phenomenon and can therefore survive in AC fields [30,31,32]. Since the electrothermal body force density increases with temperature gradient, it is efficient to make use of ACET to manipulate high-conductivity biological buffers that can even produce sufficient electric heat generation at small voltages [33,34,35]. Artificial heating elements have to be embedded into the device internal, however, in order for ACET to act effectively on dilute electrolyte, which requires intricated micro-processing techniques [36]. 

Different form ACET which appears as a kind of bulk electroconvection due to Maxwell-Wagner structural polarization, induced-charge electrokinetics (ICEK) originates from diffuse charge dynamics over ideally or even weakly polarizable surfaces driven by external DC/AC fields [37,38,39]. That is, ICEK occurs when a background electric field induces a Debye screening layer at a polarizable solid/liquid interface due to a balance between electrostatic attraction and thermal diffusion, and then forces the counterionic charge within the induced double layer (IDL) into ICEO streaming flow [40,41,42]. Since the Debye length containing mobile ions decays with increasing ionic strength [43,44,45], ICEO is particularly effective for manipulating low-conductivity liquid medium [46,47,48,49,50,51]. 

To our best knowledge, field-effect flow control is most initially proposed for accelerating traditional DCEO pump flow [52,53,54]. In this study, however, we innovatively introduce this concept to the field of nonlinear electrokinetics. Specifically, AC field-effect control through a gate terminal immersed in buffer solution enables ICEO to generate arbitrary symmetry breaking in flow pattern of induction whirlpools. This idea of field-effect control on nonlinear electroosmosis is vividly referred to as ‘AC flow field-effect transistor’ (AC-flow-FET) [55,56]. With appropriate device architecture, asymmetric ICEO micro-vortices resulted from biased gate voltages in AC-flow-FET would bring great benefits to sample mixing in dilute electrolyte, as is the major focus of subsequent analysis.

## 2. Materials and Methods

### 2.1. Device Design of Micromixers with AC Field-Effect Flow Control on ICEO

**Figure 1:**
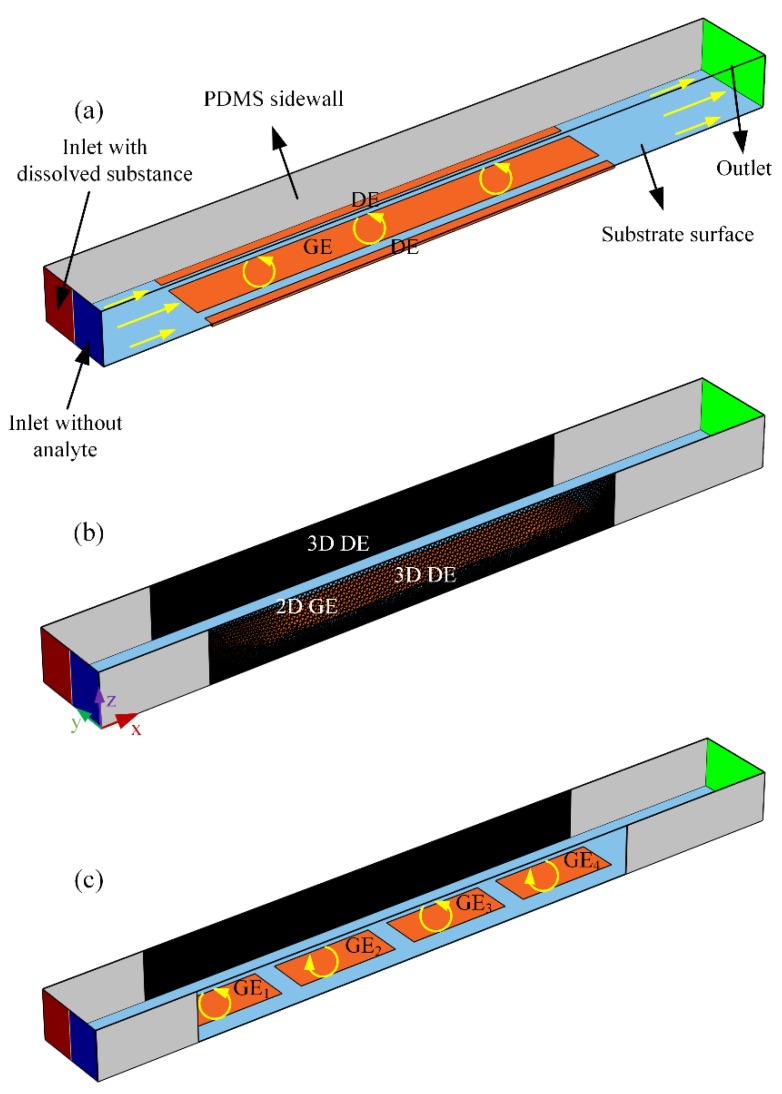
Device design of field-effect-reconfigurable ICEO micromixer with the configuration of three parallel metal strips. (**a**) An AC-flow-FET micromixer using 2D electrode structures, in which the co-flowing laminar streams intersect perpendicularly with the transversal asymmetric ICEO whirlpool, rending the two-phase contact interface rotate in counterclockwise direction on the surface of the central GE strip, helping mingle the analyte carried by the incoming pressure-driven flow to certain extent; (**b**) An ICEO micromixer in 3D electrode configurations, in which a pair of 3D sidewall DE is in perpendicular orientation to the flat GE metal strips placed along the centerline of channel bottom surface, this can greatly enhance the device performance compared with its 2D counterpart (**a**), taking into account the vanishing ACEO on DE and enhanced double-layer polarization on GE. (**c**) An integrated high-throughput ICEO micromixer with 3D electrode layouts, where four GE are disposed sequentially along the channel length direction, and controlled by two oppositely polarized gate terminals. It is noteworthy that the rotating direction of ICEO micro-eddy on each individual GE can be arbitrarily adjusted by applying different gate polarity, so as to spin and enlarge the diffusing phase boundary more efficiently and lead to better mixing performance than that of only having one gate terminal (**b**).

On the basis of field-effect nonlinear electroosmosis control, we then attempt to develop active electrokinetic micromixers of optimal performance for dilute fluids. The basic structure of the micromixer using AC-flow-FET is schematically exhibited in Figure 1a,b. The microdevice has three branch channels, including two inlets for flow injection and a single outlet for sample discharge, in correspondence to three reservoirs, respectively. The entrance of the microchannel is of a ‘Y’ structure (not shown).

Two distinct designs of electrode structure are conceived here. The 2D and 3D electrode configurations are shown in Figure 1a,b, respectively. The 2D case employs three ideally polarizable metal strips, including a pair of driving electrode (DE) of same length *L_D_* and a central gate electrode (GE) of length *L_G_* arranged on the surface of glass substrate in parallel along the longitudinal channel of length *L_C_*. In contrast, under 3D situation, a pair of Ag-PDMS conducting electrodes are embedded into the channel sidewalls, in perpendicular orientation with the gate strip positioned on the channel bottom surface (Figure 1b). A proper application of 3D DE can enhance double-layer polarization at the solid/electrolyte interface and thereby accelerate the turbulent electroconvection on the GE surface, which improves substantially the device performance in comparison with the 2D layout using coplanar metal bars.

For the simulation analysis of both configurations, a straight PDMS microchannel of height *H* is tightly bonded with a glass base (not shown). The GE bar at the channel centerline has a width of *W_G_*, and the 2D DE of *W_D_*, respectively, with an interelectrode separation *W_DG_* between DE and GE adjacently placed, resulting in a total span *W_C_* = 2*W_DG_* + *W_G_* + 2*W_D_* of the microchannel (Figure 1a). With 3D electrodes (Figure 1b), however, the nearest distance between the central GE and sidewall DE equals *W_DG*3*D_* = *W_D_* + *W_DG_*, such that the channel width *W_C_* would keep the same for both geometry.

In the calculation, the fluidic channel is first flushed with a buffer solution of ionic conductivity *σ* = 0.001 S/m and dielectric permittivity *ε* = 80*ε*_0_. The left inlet branch continuously injects fresh liquid of identical electrical properties, which carries the fluorescein nanoparticles for mixing, while the right entrance pumps aqueous electrolyte without colloidal particles. On account of the molecular diffusion effect, the phase boundary between the co-flowing laminar streams is slightly expanded, mingling the fluorescein to a minor degree, and a limited mixing performance lower than 20% is anticipated at the exit of the main channel.

For both device structures (Figure 1a,b), nevertheless, once we switch the multichannel function generator on, imposing a harmonic driving voltage of *V_D_*cos(2π*ft*) to the pair of DE (either 2D or 3D), and an in-phase gate voltage wave *V_G_*cos(2π*ft*) of identical exciting frequency to the central gate terminal. Here, *V_D_* and *V_G_* stand for the amplitude of driving and gate voltage signals, *f* and ω = 2π*f* the linear and angular frequency, respectively. With the help of AC field-effect flow control, a rotating ICEO vortex of adjustable flow profile would be produced on the surface of gate bars in the lateral direction, which perpendicularly intersects with the two side-by-side incoming laminar streams to result in helical particle-flow locus toward the channel exit, such that the phase interface stretches and rotates more rapidly than the situation merely exploiting molecular diffusion effect in a concentration gradient. For this reason, field-effect-reconfigurable ICEO whirlpools in the device structure of ac-flow-FET can flexibly direct the mixing behavior of incoming fluidic samples, especially with the 3D electrode layout (Figure 1b), as will be discussed in Section 3.1. 

To further witness the viability of field-effect flow control on ICEO, a paradigm of high-flux 3D micromixer using ac-flow-FET is developed, where a series of gate electrode bars rather than a single one are placed sequentially along channel length direction, as shown in Figure 1c. In this highly-integrated microdevice, as asymmetric ICEO eddies with opposite streaming directions alternate along the path of Poiseuille flow, the rotating direction of the diffusing phase interface can be arbitrarily tuned by imposing distinct ac voltage phase to each individual gate terminal. Consequently, with a moderate distance cycle for bidirectional double-layer polarization of the lateral electroosmotic flow, we can obtain better device efficiency from this advanced mixer in terms of high-throughput and optimal mixing performance (Figure 1c) than the simplified case only taking advantage of unidirectional electrochemical polarization (Figure 1b).

### 2.2. Theoretical Basis

For mathematical simulation, we can partition the entire microsystem into two correlated regions, including the IDL at the surface of ideally polarizable metal electrodes and bulk of the buffer medium. Within the latter, the liquid electrical properties are homogeneous to the leading order. The charge conservation for the AC potential is therefore governed by the Laplace equation. For analytical convenience, we introduce the complex amplitude for harmonic electric field variables, e.g., the transient potential ϕ(t)=Re(ϕ˜ejωt), in which $\tilde{\phi }$ with a tilde symbol denotes the voltage phasor in frequency domain. In this way, current continuity condition in the bulk under sinusoidal steady state becomes:

(1) $$\nabla^{2}\tilde{\phi }=0$$

Under the Debye-Huckel limit, we set aside those non-ideal effects that may suppress ICEO fluid motion, including electrode reaction, concentration polarization, steric effect and so on. The IDL around the polarizable surfaces consists of a compact Stern layer of capacitance *C_S_*, and a diffuse double-layer of capacitance $C_{D}=\varepsilon /\lambda_{D}$, and they are placed in series to sustain the entire overpotential at the electrode/medium interface. Here, $\lambda_{D}$ is the Debye length, and ε the buffer permittivity. Accordingly, the total capacity of IDL comes from the combination of the above two layers $C_{0}=C_{D}C_{S}/\left(C_{D}+C_{S}\right)=C_{D}/\left(1+\delta \right)$, with $\delta =C_{D}/C_{s}$ being the surface capacitance ratio. Since the electrode surface blocks any normal ion motion within a thin boundary layer, Ohmic current from the bulk has to relay the displacement current running across the IDL at the outer edge of Debye layer [57]:

(2) $$\sigma n\cdot \nabla \tilde{\phi }=j\omega \frac{C_{D}}{1+\delta }\left(\tilde{\phi }-{\tilde{\phi }}_{E}\right)$$

where σ denotes the medium conductivity, $\tilde{\phi }$ the complex potential in the bulk right outside the IDL, ${\tilde{\phi }}_{E}$ the voltage phasor of either the driving or gate electrode, and n the unit vector normal to the electrode surfaces. It is assumed the IDL can only be short circuited by polarization current in harmonic AC fields. We need not resolve the internal structure of Debye screening layer, and instead, surface impedance layer on the electrode surface is treated as a Robin-type boundary condition Equation (2). In this way, capacitive charging of IDL at the electrode/electrolyte interface can be numerically reconstructed. A scaling analysis of Equation (2) presents a characteristic RC relaxation frequency $f_{RC}=\left(1+\delta \right)\sigma \lambda_{D}/2\pi \varepsilon R=O(100)\;\mathrm{Hz}$ for dilute sample solution at micrometer dimension, with *R* denoting the macroscopic distance scale of electrochemical polarization, e.g., *R* = *W_G_*/2 for ICEO on GE and *R* = *W_G_* + 2*W_DG_* ≈ *W_C_* for ACEO on DE. After substitution, $f_{GE}^{RC}=\left(1\;+\;\delta \right){\sigma \lambda }_{D}/\pi \varepsilon W_{G}$ and $f_{DE}^{RC}=\left(1\;+\;\delta \right){\sigma \lambda }_{D}/2\pi \varepsilon W_{C}$, with $f_{DE}^{RC}$ always no more than $f_{GE}^{RC}$ in current device design. 

A portion of the applied voltage difference drops across the diffuse double-layer, with its phasor amplitude given by [58]:

(3) $$\tilde{\zeta }=\frac{1}{1+\delta }\left({\tilde{\phi }}_{E}-\tilde{\phi }\right)$$

Since all the electrodes are fixed in space, electrostatic force within the diffuse screening cloud gives rise to steady ICEO slip fluid motion on electrode surfaces in the presence of a tangential field component Et=Re(E˜tejωt)=Re((E˜−E˜⋅n⋅n)ejωt) according to the generalized Helmholtz formula:

(4) uslip(t)=−εζηEt=−εηRe(ζ˜ejωt)Re((E˜−E˜⋅n⋅n)ejωt)

where η is the dynamic viscosity of the liquid medium.

For analytical convenience, we take advantage of the time-averaged counterpart of Equation (4):

(5) 〈uslip(t)〉=−ε2ηRe(ζ˜⋅(E˜−E˜⋅n⋅n)*)=ε2η(1+δ)Re((ϕ˜E−ϕ˜)⋅(∇ϕ˜−∇ϕ˜⋅n⋅n)*)

where <*A*> denotes the time-average of *A*, and asterisk * the complex conjugate operator.

The time-averaged ICEO slipping $\langle u_{slip}\left(t\right)\rangle$ is inserted to the steady-state full Stokes equation by subjecting these ideally polarizable surfaces to slip-wall boundary conditions, so as to numerally describe electroconvection from AC field-effect flow control:

(6a) $$-\nabla p+\eta \nabla^{2}u=0\;$$

(6b) ∇⋅u=0 

where *p* denotes the scalar field of hydraulic pressure. 

The standard convection-diffusion equation is utilized in current analysis to obtain the concentration distribution of analyte injected from the left entrance:

(7) ∇⋅(uc−D∇c )=0

where *D* represents the thermal diffusivity, and *c* the concentration field of fluorescein samples that ought to be well mixed before performing any following analysis.

### 2.3. Numerical Simulation 

We use a commercial software package, Comsol Multiphysics (version 5.3a, COMSOL, Stockholm, Sweden), to analyze the ICEO fluid motion with field-effect flow control and its application to sample mixing in microchannels. The simulation procedure of electroosmotic flow field and convective mass transfer in the fluidic channel is as follows. Firstly, we compute the Laplace equation (Equation (1)) to get the AC potential phasor within the buffer solution. RC charging condition Equation (2) is imposed to the electrode/electrolyte interface, where ${\tilde{\phi }}_{E}$ = *V_D_*, ${\tilde{\phi }}_{E}$ = *V_G_*, and ${\tilde{\phi }}_{E}$ = 0 for the left DE, central GE and right DE, respectively, to delineate electrochemical ion relaxation within the IDL. The normal current vanishes on other insulating surfaces, i.e., $n\cdot \nabla \tilde{\phi }=0$.

Next, the full Stokes equation (Equation (6)) is solved for obtaining the synthetic flow field, incorporating an axial Poiseuille flow due to a pressure difference externally applied across the inlet and outlet of the fluidic channel, and the transversal ICEO vortex streaming on application of an ac voltage wave. On one hand, an inlet flow velocity *u*_0_ is designated at the channel entrance, and zero hydrodynamic pressure is assumed at the channel exit, so as to mimic the pressure-driven flow for downstream transport of the incoming nanoparticles along channel length direction. On the other hand, the ICEO fluid motion is embodied by inserting the time-averaged electrokinetic slip expression Equation (5) as a leaking-wall boundary condition on all the blocking electrodes, while other channel sidewalls are strictly subjected to no slip and no penetration.

Finally, the mass-transfer equation (Equation (7)) is computed in the fluidic channel, any normal flux is inhibited at all the solid/liquid interfaces. We use fluorescein spheres of 40 nm in diameter with Brownian diffusivity *D* = 10^−11^ m^2^/s as the test particles for mixing, the concentration value of which is *c* = 1 mol/m^3^ at the left inlet (red color in Figure 1) and 0 mol/m^3^ (blue part in Figure 1) at the right entrance, respectively, coinciding with the realistic situation of continuous sample mixing. In addition, normal diffusive mass transfer approaches zero at the channel exit.

Stationary solvers are employed for all the governing equations subjected to given boundary conditions. The AC potential phasor, fluid dynamics and sample delivery are solved sequentially with PARDISO algorithm, and grid-independence is scrutinized for each calculation result.

### 2.4. Development of the Mathematical Model

In a standard Cartesian coordinate system with three orthogonal axis (*x*, *y*, *z*), all the fundamental equations and boundary conditions possess explicit mathematical expressions, complying with the simulation method depicted in Section 2.3.

As for governing equations, we have:

(8) $$\frac{\partial^{2}\tilde{\phi }}{\partial x^{2}}+\frac{\partial^{2}\tilde{\phi }}{\partial y^{2}}+\frac{\partial^{2}\tilde{\phi }}{\partial z^{2}}=0$$

(9) $$\frac{\partial u_{x}\;}{\partial x}+\frac{\partial u_{y}}{\partial y}+\frac{\partial u_{z}}{\partial z}=0$$

(10) $$-\frac{\partial p\;}{\partial x}+\eta \left(\frac{\partial^{2}u_{x}}{\partial x^{2}}+\frac{\partial^{2}u_{x}}{\partial y^{2}}+\frac{\partial^{2}u_{x}}{\partial z^{2}}\right)\;=\;0$$

(11) $$-\frac{\partial p\;}{\partial y}+\eta \left(\frac{\partial^{2}u_{y}}{\partial x^{2}}+\frac{\partial^{2}u_{y}}{\partial y^{2}}+\frac{\partial^{2}u_{y}}{\partial z^{2}}\right)\;=\;0$$

(12) $$-\frac{\partial p\;}{\partial z}+\eta \left(\frac{\partial^{2}u_{z}}{\partial x^{2}}+\frac{\partial^{2}u_{z}}{\partial y^{2}}+\frac{\partial^{2}u_{z}}{\partial z^{2}}\right)\;=\;0$$

(13) $$u_{x}\frac{\partial c\;}{\partial x}+u_{y}\frac{\partial c}{\partial y}+u_{z}\frac{\partial c}{\partial z}-D\left(\frac{\partial^{2}c}{\partial x^{2}}+\frac{\partial^{2}c}{\partial y^{2}}+\frac{\partial^{2}c}{\partial z^{2}}\right)=0$$

To close the boundary-value problem, we analyze the integral form of above partial differential equations (PDE) to obtain the rational boundary condition at different structural interfaces:

At the channel inlets, we have:

(14) $$\frac{\partial \tilde{\phi }}{\partial x}n_{x}+\frac{\partial \tilde{\phi }}{\partial y}n_{y}+\frac{\partial \tilde{\phi }}{\partial z}n_{z}=0;\;u_{x}=u_{0}(y,z);\;c\;=\;c_{0}(y,\;z)$$

At the channel exit:

(15) $$\frac{\partial \tilde{\phi }}{\partial x}n_{x}+\frac{\partial \tilde{\phi }}{\partial y}n_{y}+\frac{\partial \tilde{\phi }}{\partial z}n_{z}=0;\;p\;=\;0;\;\frac{\partial c\;}{\partial x}n_{x}+\frac{\partial c}{\partial y}n_{y}+\frac{\partial c}{\partial y}n_{y}=0$$

where ***n*** = (*n_x_*, *n_y_*, *n_z_*) represents the local unit normal vector on the surface of interest.

At the electrode/electrolyte interface, including both DE and GE:

$$\sigma \left(\frac{\partial \tilde{\phi }\;}{\partial x}n_{x}+\frac{\partial \tilde{\phi }}{\partial y}n_{y}+\frac{\partial \tilde{\phi }}{\partial z}n_{z}\right)=j\omega \frac{C_{D}}{1+\delta }(\tilde{\phi }-V_{D/G});$$

ux=ε2η(1+δ )Re((ϕ˜E−ϕ˜)⋅(∂ϕ˜∂x−(∂ϕ˜∂xnx+∂ϕ˜∂yny+∂ϕ˜∂znz)⋅nx)*);

(16) uy=ε2η(1+δ)Re((ϕ˜E−ϕ˜)⋅(∂ϕ˜∂y−(∂ϕ˜∂xnx+∂ϕ˜∂yny+∂ϕ˜∂znz)⋅ny)*);

uz=ε2η(1+δ )Re((ϕ˜E−ϕ˜)⋅(∂ϕ˜∂z−(∂ϕ˜∂xnx+∂ϕ˜∂yny+∂ϕ˜∂znz)⋅nz)*);

$$\frac{\partial c\;}{\partial x}n_{x}+\frac{\partial c}{\partial y}n_{y}+\frac{\partial c}{\partial y}n_{y}=0$$

At other insulating surfaces:

(17) $$\frac{\partial \tilde{\phi }}{\partial x}n_{x}+\frac{\partial \tilde{\phi }}{\partial y}n_{y}+\frac{\partial \tilde{\phi }}{\partial z}n_{z}=0;u_{x}=u_{y}=u_{z}=0;\;\frac{\partial c\;}{\partial x}n_{x}+\frac{\partial c}{\partial y}n_{y}+\frac{\partial c}{\partial y}n_{y}=0$$

Equations (8)–(13) subjected to boundary conditions Equations (14)–(17) met the definite solution condition of boundary value problem. That is, our mathematical model invariably has a unique solution under a given set of experimental parameters.

### 2.5. Evaluation of the Mixing Performance

The mixing index γ can correctly quantify the device performance once the concentration distribution of nanoparticle samples at the channel outlet is known:

(18) $$\begin{array}{l} \gamma =\left(1-\frac{\iint_{S}\left|c-0.5[\mathrm{mol}/m^{3}]\;\right|dA}{\iint_{S}0.5[\mathrm{mol}/m^{3}]dA}\right)\times 100\%\\ =\left(1-\frac{\int_{0<z<H}\int_{0<y<W_{C}}\left|c-0.5[\mathrm{mol}/m^{3}]\right|dydz}{\int_{0<z<H}\int_{0<y<W_{C}}0.5[\mathrm{mol}/m^{3}]dydz}\right)\times 100\% \end{array}$$

In this equation, the area integral performed on the channel exit *S* is transformed into a double integral, since d*A* = d*y*d*z* for a plane of interest that is perpendicular to the *x* axis. 

## 3. Results and Discussion

### 3.1. A Comparative Study of ICEO Micromixers with 2D and 3D Electrode Layouts

**Figure 2:**
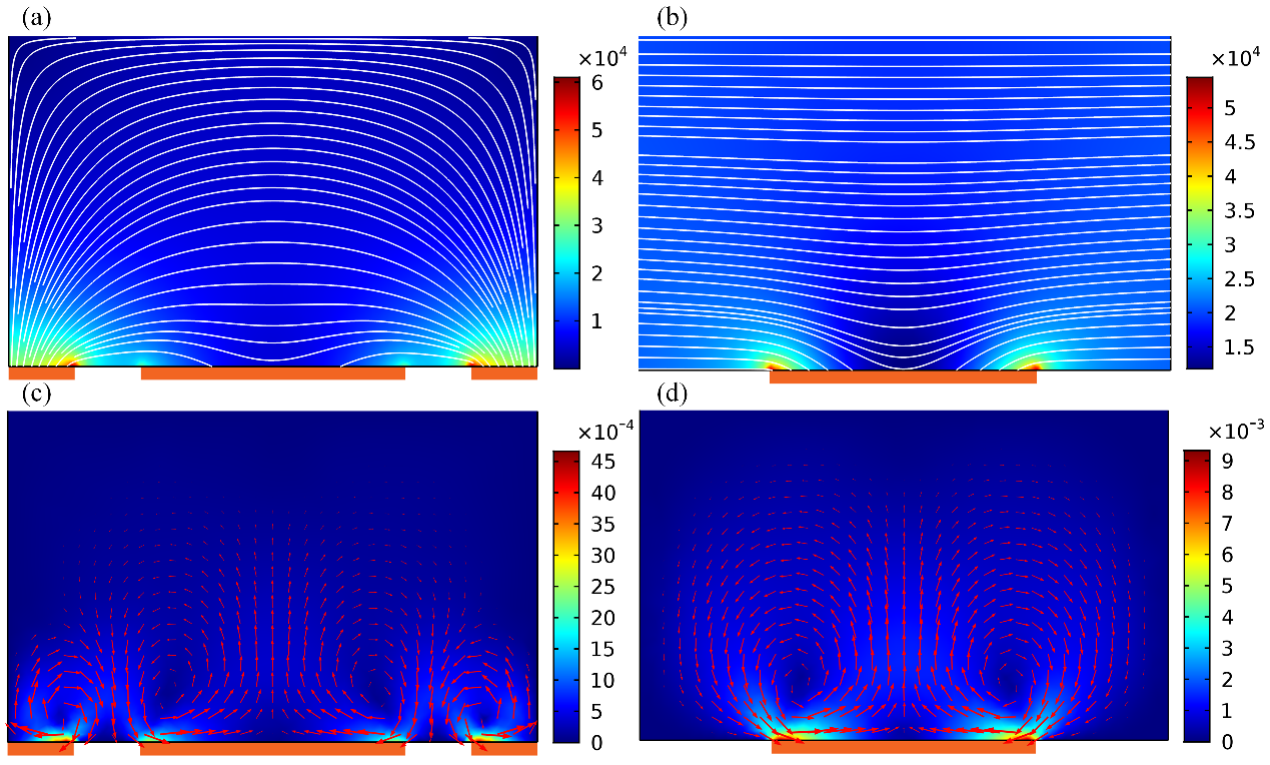
Under identical electric field intensity, a comparison of the basic trait of unbiased ICEO eddies above one central GE in the flow-FET configuration with 2D and 3D driving electrodes at given field frequency *f* = 200 Hz. For 3D electrode layouts in (**b**,**d**), *V_D_* = 2*V_G_* = 8 V; while for the 2D counterpart in (**a**,**c**), *V_D_* = 2*V_G_* = 6 V, such that they share a same magnitude of background electric field of *E_b_* = 20,000 V/m. (**a**) A surface and arrow plot of electric field distribution (unit: V/m) in the 2D, and (**b**) 3D electrokinetic micromixer. (**c**) Transversal ICEO fluid motion (unit: m/s) with GE floating in the 2D, and (**d**) 3D device design.

**Figure 3:**
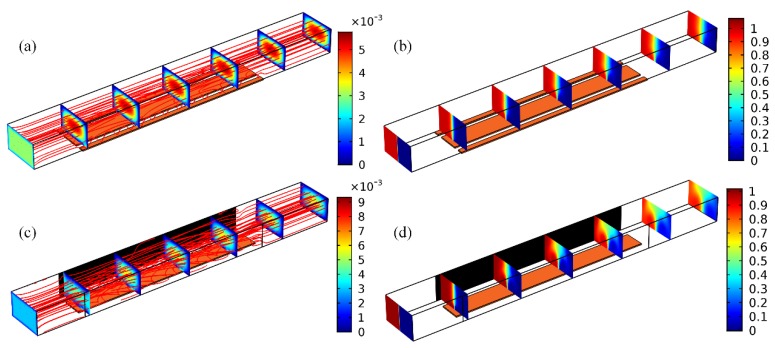
Simulation result of ICEO sample mixing in axial continuous flow of inlet flow rate *u*_0_ = 3mm/s and solution conductivity 0.001 S/m in the fundamental configuration of single floating gate terminal under given frequency *f* = 200 Hz and field intensity *E_B_* = 20,000 V/m. (**a**,**b**) With 2D electrode layouts, symmetrically circulating ICEO vortex flow induced on the surface of gate strip intersects perpendicularly with the incoming sample stream, resulting in a mixing performance of *γ* = 26.48% by lateral electroconvection which extends the phase interface without any hydrodynamic torque, (**c**) a streamline and multi-slice surface plot of flow field (unit: m/s), (**d**) a multi-slice surface plot of analyte concentration distribution affected by symmetric electroosmotic whirlpools in the transverse plane (unit: mol/m^3^). (**c**,**d**) Corresponding simulation results in 3D electrode configuration, with an enhanced device efficiency of *γ* = 50.1%, albeit still not ideal.

An investigation on ICEO streaming reconfigurable through field-effect flow control may provide a theoretical foundation for developing high-efficiency active micromixers. At the very beginning, we focus on the traditional ICEO vortex flow pattern with central GE free from external wiring, i.e., *V_G_* = *V_D_*/2. A suitable parametric space is selected for the numerical simulation: *W_C_* = 400 μm, *W_D_* = 50 μm, *W_G_* = 200 μm. *W_DG_* = 50 μm, *f* = 200 Hz, *L_D_* = *L_G_* = 2 mm, *L_C_* = 3.3 mm, *H* = 250 μm. To make a fair comparison between micromixers of 2D (Figure 1a) and 3D electrode layouts (Figure 1b), *V_D_* = 6 V and *V_D_* = 8 V are imposed to the 2D (Figure 2a,c) and 3D DE pair (Figure 2b,d), respectively.

In ICEO, a background electric field acts on its own induced Debye screening charge within the EDL, resulting in time-averaged electroosmotic streaming on ideally polarizable surfaces even in oscillating AC fields (Figure 2). In the early stage, the buffer solution is supposed to have an ionic strength of 0.001 S/m, resulting in a bulk charge relaxation frequency *f*_bulk_ = σ/2πε = 225 kHz. In the device of 2D electrodes, strong electric field is produced at the corner-field-singularity of DE on both sides, indirectly weakening the potential gradient across the planar surface of GE strip (Figure 2a). On application of a low-frequency ac signal, ICEO streaming appears and behaves as two pairs of counter-rotating micro-vortices above the three parallel electrode bars (Figure 2c). Focusing on one of the electrode spacings, ICEO vortex flow field resembles ACEO induced on a single coplanar electrode pair. That is, ICEO whirlpool in 2D flow-transistor cascades within interelectrode gaps, sweeps across the ideally polarizable surface, then streams upward, giving rise to four recirculating fluid loops (Figure 2c). 

In stark contrast, as to the 3D layout Figure 2b,d, a relatively large area of the sidewall DE pair makes the electric field distribution much more uniform, and a local maximum intensity exists in the immediate vicinity of two edges of GE (Figure 2b), rather than the corner of DE pair in 2D configuration (Figure 2a). At the price of same energy dissipation, the usage of 3D sidewall electrodes enhances electrochemical polarization at GE/electrolyte interface (Figure 2b), and therefore produces stronger ICEO fluid motion inside the microchamber (Figure 2d). For this special device design, however, it is not possible for the ACEO convection to occur with no tangential field component on the DE surface, so that there is merely one pair of counter-rotating ICEO micro-vortices on GE along the transversal direction (Figure 2d). Even so, both the flow velocity and actuating range of ICEO vortex are greatly enhanced by exploiting 3D sidewall DE (Figure 2d) in comparison to coplanar arrangement (Figure 2c). 

Above results about electro-convective streaming suggests that the 3D device design (Figure 1b) is of more superiority in developing high-performance micromixer than its 2D counterpart (Figure 1a). We then need to make a test validation on this inference. As shown Figure 3, the incoming pressure-driven laminar stream has a parabolic profile, with flow velocity of *u*_0_ = 3 mm/s at the channel entrance. Without external powering, any mixing effect in a straight fluidic channel is in essence caused by molecular diffusion across the phase boundary. At the channel exit, the vertical contact interface stretches little in the absence of lateral perturbation, generating a very poor mixing efficiency *γ* = 22.83% (not shown). On switching the signal generator on, with the GE strip floating, symmetric ICEO vortex flow pattern appears on the surface of GE along channel width direction, and the lateral electro-convective perturbation intersects perpendicularly the axial Poiseuille flow, giving rise to rotating streamlines on top of the metal strips (Figure 3a,c). As a result, not only does thermal diffusion takes effect, but also transversal electroconvection of the nanoparticle samples contributes to expanding the phase boundary of a sharp concentration gradient (Figure 3b,d). Because of a higher efficiency in electromechanical energy conversion, as has been demonstrated in Figure 2, the 3D device design (Figure 3d) performs much better, in terms of producing a higher mixing performance *γ* = 50.1% than the 2D electrode configuration (Figure 3b) with *γ* = 26.48%. 

Though the contact interface is simultaneously extruded by molecular diffusion and circulatory ICEO streamlines, it does not undergo any rotating motion because of the geometric symmetry in field-induced Debye screening on GE surface even in 3D electrode configuration. Since the rate of substance exchange across the phase boundary has a limitation without electrohydrodynamic torques, sample mixing above a floating gate is still unsatisfactory using sidewall DE (Figure 3d). Achieving further improvement through the AC field-effect control on ICEO is urgently needed.

### 3.2. Mixing with AC Field-Effect Flow Control in the 3D Device Design

It has been demonstrated that the 3D electrode layout is an improved device design in terms of developing microfluidic mixers. In this sense, unless otherwise noted, all the subsequent analysis is concentrated on the 3D microdevice. When the central GE is exempt from external wiring (Figure 2b), electroosmotic eddies in reverse rotating directions encounter and counterbalance one another at the center of GE surface, developing an in-situ flow stagnation line (FSL) (Figure 2d). Symmetrically-distributed stagnation areas can be fully exploited for preconcentration of colloidal particles, but are not efficient in steering the motion of two-phase contact interface for sample stirring. 

**Figure 4:**
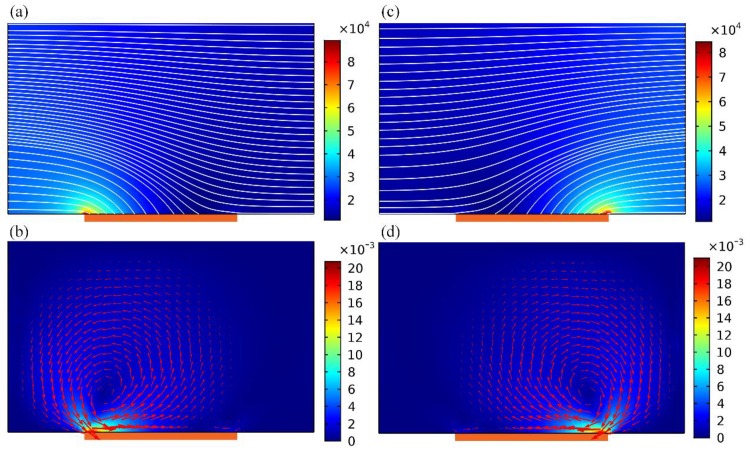
Simulation result of AC field-effect control on ICEO with non-neutral gate voltages in 3D electrode configurations, i.e., *V_G_* ≠ *V_D_*/2 = 4 V, reconfigurable vortex flow pattern can be induced across channel width direction. (**a**,**b**) When the gate polarity is negative *V_G_* < *V_D_*/2 (*V_D_* = 8 V, *V_G_* = 2 V), electrochemical polarization becomes more significant adjacent to the left side of gate terminal, giving rise to a single large-scale ICEO vortex flow rotating counterclockwise, (**a**) a surface and streamline plot of the asymmetric electric field distribution (unit: V/m), (**b**) a surface and arrow plot of ICEO flow field, in which an anticlockwise eddy overwhelms the fluid motion within the entire fluidic channel (unit: m/s). (**c**,**d**) As GE is positively polarized *V_G_* > *V_D_*/2 (*V_D_* = 8 V, *V_G_* = 6 V), gradients of both the electric field and flow field variables reverse in the horizontal direction with respect to (**a**,**b**), respectively. Most importantly, the rotating direction of ICEO vortex is inverted, and the clockwise electroosmotic micro-vortex near the right rim of GE governs the transverse electroconvection.

Prompted by the previous work on field-effect flow control in microfluidic networks [53], a biased gate voltage *V_G_* can help adjust electro-convective mass transport along channel transverse direction. As exhibited in Figure 4a,c, corresponding to a negatively- or positively- polarized gate terminal, the electric field intensity augments on the left or right side of gate strip surface, but decreases on the other side. This directly results in asymmetric distribution of the electrostatic potential gradient, which interplays with enhanced electrochemical polarization at the same site to induce biased ICEO vortex flow field (Figure 4b,d). The flow velocity and rotating direction can be arbitrarily reconfigured by adjusting the gate voltage amplitude *V_G_*. For this reason, AC field-effect control on ICEO circulation opens up new opportunities for handling nanoscale entities in dilute electrolyte in the context of microfluidics.

**Figure 5:**
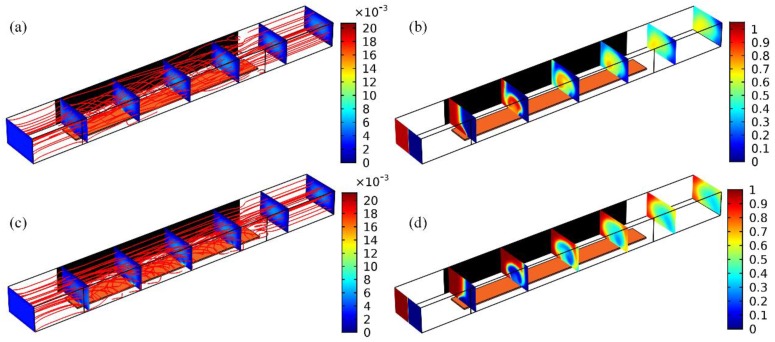
Calculation result of sample mixing behavior via field-effect-reconfigurable ICEO vortex flow field under distinct gate polarities and a fixed driving voltage *V_D_* = 8 V, the device performance is improved greatly, taking into consideration an additional electrohydrodynamic torque exerted on the phase boundary. (**a**,**b**) Nanoparticle mixing reinforced by a single dominating ICEO vortex with anticlockwise recirculation as the gate voltage *V_G_* = 2 V is lower than the balance potential *V_D_*/2 = 4 V (*V_G_* − *V_D_*/2 = −2 V, negatively polarized GE), with mixing performance *γ* = 76.1%, (**a**) a streamline and cross-sectional magnitude plot of the helical flow field rolling counterclockwise (unit: m/s), (**b**) a multi-slice magnitude plot of sample concentration distribution (unit: mol/m^3^). (**c**,**d**) Mixing behavior with clockwise ICEO vortex as the gate voltage *V_G_* = 6 V is beyond the neutral one *V_D_*/2 = 4 V (*V_G_* − *V_D_*/2 = 2 V, positive gate polarity), with same device efficiency *γ* = 76.1% owing to a mirror image of the gate voltage offset, (**c**) a streamline and cross-sectional magnitude plot of the helical streamlines in clockwise rotating direction (unit: m/s), (**d**) a multi-slice surface plot of nanoparticle concentration distribution (unit: mol/m^3^).

As shown in Figure 5, by imposing a sufficiently large offset voltage $\left|V_{G}-0.5V_{D}\right|$ to the gate terminal, biased ICEO whirlpool twirling in either anticlockwise (Figure 5a, for negative gate polarity) or clockwise direction (Figure 5c, for positively-polarized GE) can be generated above the metal strip. The asymmetric electroosmotic vortex flow exerts a time-averaged electrohydrodynamic torque on the buffer solution, rendering the phase boundary revolve in pace with the transversal electrokinetic circulation. The additional rotating motion twists and expands the two-phase contact interface in a more effective manner than the unbiased situation for an identical longitudinal distance. So, swapping of substance between the co-flowing buffer streams is sped up, irrespective of the rotating direction of ICEO vortex flow field (Figure 5b,d), leading to an elevated mixing efficiency *γ* = 76.1% compared to the symmetric configuration of *γ* = 50.1%. The incoming laminar streams intersect perpendicularly with the asymmetric ICEO eddy, resulting in helical streamlines rolling forward above the GE surface (Figure 5a,c). Although the forward helix in Figure 5a,c rotates in opposite directions, however, there is not a clear difference in mixing of nanoparticles on account of symmetry in the magnitude of gate voltage offset. 

### 3.3. Effect of Different Experimental Parameters on the 3D ICEO Micromixer

Based upon the above simulation studies, asymmetric ICEO whirlpool is more efficient in engendering active mixing in microfluidics than the symmetric case in which the central gate terminal floats in potential. It is then essential to elucidate how the different experimental parameters including the harmonic frequency, gate potential offset and inlet flow velocity, can exert an impact on device performance of the 3D ICEO micromixer

#### 3.3.1. Frequency-Dependence 

Since both DE and FE undergo electrode polarization that is strongly affected by electrochemical ion relaxation in alternating fields, the frequency-dependence of ICEO flow velocity with field-effect control must be quite complex. In DC limit, with the field frequency much lower than the inverse double-layer relaxation time on DE pair, i.e., $f\ll f_{DE}^{RC}=\left(1+\delta \right){\sigma \lambda }_{D}/{2\pi \varepsilon W}_{C}$, most of the applied AC voltage drops across the IDL at the DE/electrolyte interface due to complete Debye screening, leaving no electric field in the bulk to force the mobile ions into ICEO streaming. For frequency well beyond the characteristic relaxation frequency of the blocking surface of GE, i.e., $f\gg f_{GE}^{RC}=\left(1+\delta \right){\sigma \lambda }_{D}/{\pi \varepsilon W}_{G}$, there is not enough time for the counterionic charge to accumulate in the IDL at the GE/electrolyte interface within each harmonic cycle, resulting in null ICEO fluid motion once again due to incomplete Debye screening on GE. So, the best mixing behavior should take place in the intermediate frequency range, i.e., $f_{DE}^{RC}<f_{\mathrm{ideal}}<f_{GE}^{RC}$. 

**Figure 6:**
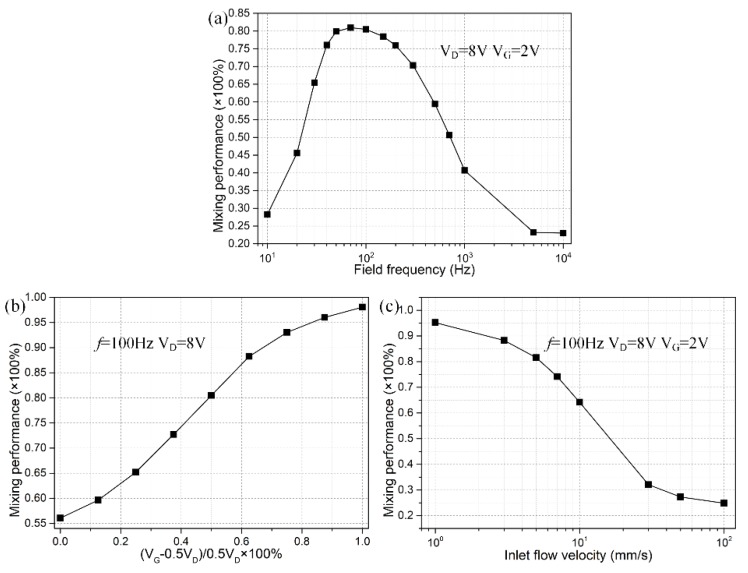
A parametric study of the 3D ICEO micromixer with ac field-effect control of lateral ICEO convection. (**a**) Frequency-dependence of mixing performance, for *V_D_* = 8 V and *V_G_* = 2 V; (**b**) Effect of gate potential offset on the device mixing behavior at frequency *f* = 100 Hz and driving voltage *V_D_* = 8 V; (**c**) Effect of inlet flow rate on the mixing efficiency under given frequency *f* = 100 Hz, driving voltage *V_D_* = 8 V, and gate voltage *V_G_* = 1.5 V, being equivalent to an offset ratio of 0.625.

As indicated by the calculation results in Figure 6a with a positive gate polarity chosen in priority, there is single peak of mixing performance of *γ* = 80% around *f* = 100 Hz, either increase or decrease the field frequency would abate the device efficiency, in good accordance with this simple physical argument. Besides, the worst performance is lower than 25%, implying that sample mixing is chiefly induced by diffusive mass transfer for *f* ≤ 10 Hz and *f* ≥ 5 kHz. As a consequence, to acquire perfect mixing behavior with AC field-effect control of ICEO, we should carefully seek the ideal operation frequency, with reference to the reciprocal double-layer relaxation time of both DE and GE, which has a strong dependence on the ionic strength of the buffer medium. 

#### 3.3.2. Influence of the Gate Voltage Offset 

For practical applications, it is of great importance to quantify how the specific value of gate potential offset affects field-effect-reconfigurable ICEO mixing at a given field frequency, e.g., *f* = 100 Hz in current device geometry. With the definition of absolute offset voltage *V_G_* − 0.5*V_D_*, a nondimensional mathematical expression $\left|V_{G}-0.5V_{D}\right|/0.5V_{D}$ is extractable, and its value is bounded between zero and unit in *real situations* where 0 ≤ *V_G_* ≤ *V_D_*. When the offset ratio $\left|V_{G}-0.5V_{D}\right|/0.5V_{D}$ of gate potential becomes zero, the GE strip floats in external fields and no symmetry breaking in ICEO vortex flow pattern takes place. As the value of $\left|V_{G}-0.5V_{D}\right|/0.5V_{D}$ approaches one, the voltage offset reaches the peak magnitude for *V_G_* = *V_D_* or *V_G_* = 0, resulting in the largest electroosmotic whirlpool regardless of the specific rotating direction. So, the value of $\left|V_{G}-0.5V_{D}\right|/0.5V_{D}$ is able to correctly evaluate the extent of symmetry breaking in transversal electroconvection. Theoretically, as $\left|V_{G}-0.5V_{D}\right|/0.5V_{D}$ becomes larger, AC field-effect control on ICEO becomes more evident and thereby ICEO vortex has a propensity to roll in a preferential direction, as demonstrated by the increasing trend of field-effect-reconfigurable device performance with $\left|V_{G}-0.5V_{D}\right|/0.5V_{D}$ (Figure 6b). The best mixing efficiency emerges at the largest offset voltage, in which the most asymmetric ICEO vortex exerts the most potent electro-rotational torque on the fluidic sample, but this would take a lot of energy. Accordingly, application of a moderate gate potential offset, which can well enhance convective mixing at no cost of severe power dissipation, ought to be our first choice. For this reason, a specific offset ratio $\left|V_{G}-0.5V_{D}\right|/0.5V_{D}\;=\;0.625$ is chosen for further theoretical studies (*V_G_* = 1.5 V for negative and 6.5 V for positive gate polarity with *V_D_* = 8 V). 

#### 3.3.3. Effect of Inlet Flow Rate

A square crossing of the axial laminar streams and lateral ICEO vortex on top of GE gives rise to forward helical streamlines, which is responsible for improving the mixing dynamics in the fluidic channel. For this reason, the flow velocity at the channel entrance fiercely influences the device efficiency. When the inlet flow velocity is slow and the Reynolds number is small, there is sufficient time for the incoming colloidal particles to get well mixed along the channel length direction as they are slowly delivered toward the outlet, producing a high mixing performance while greatly sacrificing the sample flux. On the contrary, at a high inlet flow rate, there is almost no chance for the helical streamlines to rotate a circle on the surface of gate terminal, and consequently the mixing dynamics is suppressed but high-throughput is obtainable at the channel exit, as verified by the calculation result in Figure 6c where the mixing efficiency declines as the inlet flow velocity increases. 

Then, we have to address the issue about how to mingle fluidic samples efficiently without loss of throughput. 

### 3.4. 3D High-Throughput Mixing with AC Field-Effect Flow Control

To accomplish sample mixing at a relatively large Reynolds number, electroconvection in the lateral direction must be promoted to compete against the axial pressure-driven flow. A scaling analysis of Equation (16) indicates the flow velocity of ICEO abides by a general scaling law:

(19) $$\left|u_{y}^{\mathrm{ICEO}}\right|\approx C\cdot \frac{\varepsilon V_{D}^{2}W_{G}}{4\eta \left(1+\delta \right)W_{C}^{2}\left(1+{\left(\omega \tau_{RC}\right)}^{2}\right)}$$

where *C* is a geometry-dependent prefactor, and $\tau_{RC}=\varepsilon W_{G}/2\left(1+\delta \right){\sigma \lambda }_{D}$ the RC charge relaxation time of interfacial capacitance. From Equation (19), once all the geometric and physicochemical properties are preset values, it is possible to improve the device performance by adjusting the applied voltage *V_D_* and chip structure *C*. Accordingly, we then focus on AC field-effect nonlinear electroosmosis control for high-throughput mixing, in terms of adjusting either the background field intensity or the discrete layouts of central GE strips.

#### 3.4.1. Voltage-Dependence 

**Figure 7:**
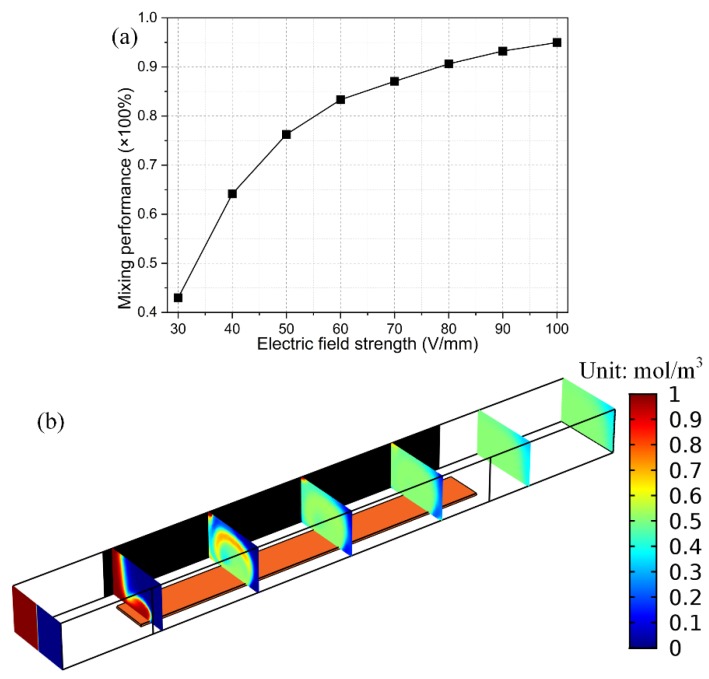
Effect of the imposed voltage magnitude on the device mixing efficiency under given voltage offset ratio 0.625, inlet flow velocity 10 mm/s, and field frequency 100 Hz. (**a**) Voltage-dependence of the mixing performance; (**b**) High-throughput mixing at *V_D_* = 20 V and *V_G_* = 3.75 V, while there is still a dead zone of ICEO turbulence on the upper right of the channel cross section.

According to Equation (19), the transversal electrokinetic turbulence is linearly proportional to the input voltage squared, implying it is a utilitarian method to rectify the mixing behavior by just increasing the magnitude of the background field strength *E_B_* = *V_D_*/*W_C_*, as shown in Figure 7a with a given gate voltage offset ratio of 0.625, inlet flow velocity of 10 mm/s, and signal frequency of 100 Hz. Besides, it is noteworthy that for the peak voltage *V_D_* = 20 V, the electrical field intensity reaches 20 [V]/200 [μm] = 100 [V/mm] within the electrode spacing. This order of magnitude of driving voltage indicates there is a potential drop of 1V across a gap of 10 μm in size, which is no less than the common situation of 0.5–1.5 V used in experimental observation of ACEO. In addition, there is always a dead zone of ICEO vortex flow field at the upper left (or upper right, Figure 7b) of the channel cross section with a unipolar gate terminal, which is positively (or negatively) polarized.

#### 3.4.2. Integrated 3D ICEO Micromixer with Bipolar Gate Terminals

**Figure 8:**
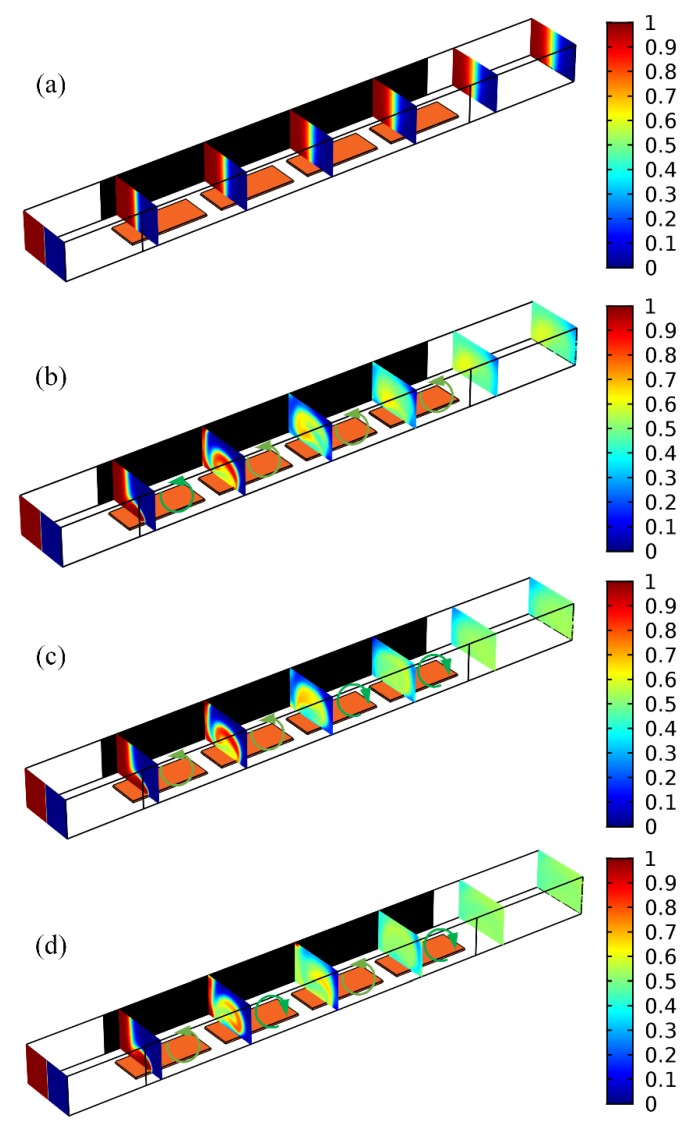
At an inlet flow velocity *u*_0_ = 10 mm/s and ion conductivity 0.001 S/m, a simulation study on the influence of different combinations of ICEO vortex polarity on mixing improvement in the integrated tandem device structure with four sequential GE strips subjected to various of gate voltage sequence. (**a**) In the absence of external powering, the phase boundary between the co-flowing laminar streams is stretched only by diffusive mass transfer with *γ* = 23.54%. (**b**,**c**) For *V_D_* = 14 V, (**b**) and *V_G_*_1_ = *V_G_*_2_ = *V_G_*_3_ = *V_G_*_4_ = 2.625 V are imposed on the four sequential GE, respectively, ICEO vortex in anticlockwise direction appears on the ideally polarizable surface of all the GE strips, which twists and stretches the phase interface in synergy with mass diffusion, resulting in an nonideal device performance of *γ* = 88.67%, (**c**) with a given gate potential sequence of *V_G_*_1_ = *V_G_*_2_ = 2.625 V and *V_G_*_3_ = *V_G_*_4_ = 11.375 V, ICEO eddy rotates anticlockwise on the surface of two GE upstream, but polarity of the electroosmotic vortex makes a reversal on the two GE downstream, so the distance scale of electrochemical polarization equals two gate length, and the transformation in rolling direction of the phase boundary at such a longitudinal displacement leads to better mixing efficiency *γ* = 92.41% than (**b**); (**d**) An more frequent alternation in flow direction of lateral ICEO vortex at a shorter distance (one GE length) gives rise to even more superior device performance *γ* = 95.233% than (**c**).

To enlarge the actuating range of ICEO mixing streamlines, we propose a more advanced discrete electrode layouts for field-effect-reconfigurable sample mixing, as shown in Figure 8. In this integrated device design, an array of GE with four coplanar metal bars subjected to different gate voltage polarities is arranged consecutively along the centerline of the insulating substrate surface. To further miniaturize the device, the length of all the GE strips is reduced to *L_G_* = 500 μm, and the separation between adjacent GE is *W_GG_* = 100 μm. The ion conductivity of the suspension medium is 0.001 S/m, low enough to evade any steric effect. Because of a decrease in the dead zone of transversal electroconvection, an AC voltage signal of moderate amplitude *V_D_* = 14 V at *f* = 100 Hz is imposed to the 3D sidewall DE. The four sequential GE strips are labelled as *G*_1_, *G*_2_, *G*_3_ and *G*_4_, and are imposed with gate potentials of *V_G_*_1_, *V_G_*_2_, *V_G_*_3_ and *V_G_*_4_, respectively. We herein utilize two opposite gate polarities for convenience of simulation analysis, *V_Gi_* = 11.375 V for the positive gate bias “+”, and *V_Gi_* = 2.625 V for the negative counterpart “−”, where 1 ≤ *i* ≤ 4 represents the *i*-th gate electrode from upstream to downstream. To test the feasibility of the integrated device structure in high-throughput sample mixing, the inlet flow velocity is enhanced to *u*_0_ = 10 mm/s. 

Without input of external electrical energy to the microdevice, there is no ICEO turbulence for electro-convective mixing, and the sole action of molecular diffusion produces a poor mixing efficiency of *γ* = 23.32% even using an GE array (Figure 8a and Table 1). On switching the multichannel function generator on, as the four GE in the strip array share an identical polarity, e.g., with a gate potential sequence −/−/−/−, anticlockwise ICEO micro-vortices of consistent flow direction appear on the ideally polarizable surface of the four GE, which convectively stretch and twist the phase boundary in synergy with diffusive mass transfer, giving rise to a modest device performance of *γ* = 88.67% (Figure 8b). 

If we make use of two gate terminals of opposite polarities rather than a single, although there are numerous combinations of vortex-flow direction, two symmetric powering conditions are studied preferentially, as shown in Figure 8c,d, since they can best represent AC field-effect control on ICEO sample mixing. With a potential sequence −/−/+/+ of two reversed gate polarities, anticlockwise and clockwise ICEO eddies are produced above the upstream and downstream two gate strips, respectively (Figure 8c). In other words, electro-convective fluid motion reverses in lateral flow direction at the downstream end of the 2nd GE, which alters the direction of rotation of the two-phase contact interface on a distance scale of double GE length, leading to an improved mixing efficiency *γ* = 92.41% from double gate polarity (Figure 8c) than *γ* = 88.67% with unipolar gate terminal (Figure 8b). 

This astonishing result encourages us to adjust the polarity of electrochemical polarization with an even shorter repeating length, as shown in Figure 8d, where the specific sequence of gate potential −/+/−/+ is imposed on the GE array. In this situation, the circulatory ICEO whirlpool changes the sense of rotation even more frequently (Figure 8d) than the “−/−/+/+” condition (Figure 8c), and inversion of electroconvection takes place on every adjacent GE of reversed gate polarity, further boosting the device performance to 95.233% (Figure 8d). To our best knowledge, an optimum distance scale in reverse of electrode polarization always exists in practical experiment, which should be the longitudinal displacement need for the phase boundary to rotate an entire circle of 360° as the fluidic samples are transported downstream. Accordingly, we can optimize field-effect-reconfigurable ICEO mixing by pursuing the best gate voltage sequence for a preset GE array in the integrated 3D high-throughput micromixer, which could help diminish the dead zones of transverse ICEO turbulence to great extent (Figure 8d).

#### 3.4.3. Conductivity-Dependence of the Integrated Micromixer

**Figure 9:**
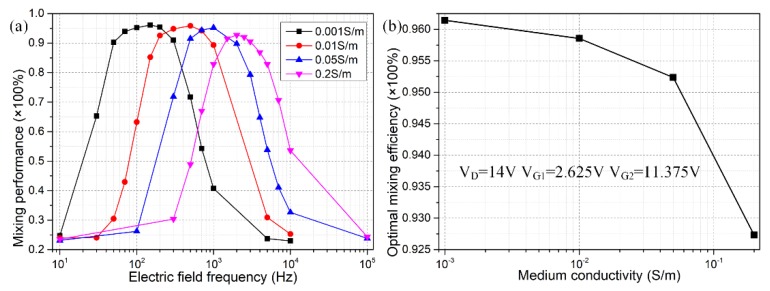
Effect of liquid conductivity on mixing efficiency of the integrated micromixer. (**a**) Frequency-dependent mixing performance for different ionic strength; (**b**) Conductivity-dependent optimum mixing efficiency at corresponding ideal operation frequencies.

The simulation analysis above has mainly concentrated on the influence of a variety of electric field parameters and the discrete electrode layout on the mixing dynamics due to AC field-effect control on ICEO. Nevertheless, medium conductivity is also a pivotal factor that greatly affects the functionality of sample mixing in the 3D micromixer. Generally speaking, a decrease in solution conductivity can extend the thickness of IDL, so the diffuse layer becomes much more important than the Stern layer in dilute electrolyte, resulting in an enhancement of ICEO flow velocity for convective mixing in low-conductivity suspension. In this way, the optimum mixing performance boosts with decreasing liquid conductivity (Figure 9b).

At the same time, the rise in ionic strength increases the double-layer relaxation frequency, since the IDL capacitance and resistor of the bulk fluid are connected in series to comprise the whole electric circuit system. The ideal operating frequency of the micromixer *f*_ideal_, which is bounded between $f_{DE}^{RC}$ and $f_{GE}^{RC}$, then makes a change for different solution conductivities. Specifically, the ideal frequency *f*_ideal_ increases from 100 Hz to 2 KHz, as the ionic conductivity grows from 0.001 S/m to 0.2 S/m (Figure 9a), while the mixing efficiency at corresponding peak frequencies decays due to a shrink in Debye screening length. For analytical convenience, however, we have disregarded the possible action of nonlinear diffuse charge dynamics beyond the Debye-Huckel limit. In fact, when the medium conductivity surpasses a threshold value on the order of O(0.01) S/m, ionic species would overcrowd inside the IDL at the electrode/electrolyte interface, and this steric effect severely exacerbates as the ion concentration further increases, which is prone to evaporate the fluid motion of ICEO with ionic strength exceeding ~0.05 S/m. As a consequence, the integrated 3D device design is eligible for convective mixing of dilute electrolyte at the micrometer dimension. 

## 4. Conclusions

In summary, we have put forward the physical concept of AC field-effect flow control on nonlinear electroosmosis, where arbitrary symmetry breaking in transversal ICEO vortex flow pattern can be realized via adjusting the AC voltage imposed to the central gate terminal immersed in buffer solution. A rigid mathematical model is established in the framework of 3D Cartesian coordinate system to study the feasibility of this unique device structure for field-effect electro-convective mixing in straight microchannels, and the earliest comparison studies indicate the 3D electrode configuration with arrangement of sidewall DE is able to achieve better field-effect mixing control than its 2D counterpart using coplanar metal strips in microsystems. The effect of various experimental parameters, such as the driving frequency, ratio of gate voltage offset and inlet flow velocity, on the performance of this 3D micromixer is investigated by direct numerical modeling. To suppress the dead zone of ICEO turbulence within the channel cross section, an integrated 3D micromixer with high-throughput is developed by employing an array of bipolar GE, in which ICEO vortex streaming on the surface of each GE can be separately addressed. With double gate terminals of reverse polarities, alternation in circulating direction of ICEO whirlpool at modest space intervals can rotate and extend the diffusing phase boundary more intensively, giving rise to even better mixing performance in the context of dilute electrolyte. The mathematical model of AC field-effect mixing control developed in this work is merely valid under Debye-Huckel approximation, and the action of nonlinear diffuse charge dynamics is excluded in this work. On account of this limitation, possible extensions of current work may include the investigation of using more complex electrode structures for controlling the field-effect-tunable mixing behavior and the coupling of asymmetric electrode polarization with effects of a relatively large Dukhin number, including bipolar Faradaic reactions, nonlinear surface capacitance, non-uniform surface conduction, ion concentration polarization, steric effect and so on. Under such situations, the linear asymptotic analysis applied herein would malfunction, and alternative mathematical models are pursued to account for the influence of nonlinear diffuse charge dynamics on AC field-effect mixing control. We believe that AC-flow-FET would inspire interdisciplinary research focusing on condensed matter, electrokinetic phenomena, and micro/nanofluidics in the near future.

## Figures and Tables

**Table 1:** Device efficiency for the integrated 3D high-throughput micromixer (Figure 8) with the central GE array subjected to different gate voltage sequence.

Specific Gate Voltage Sequence for the Ge Array	Mixing Performance
No Electric Field Supplied	23.54%
−/−/−/−	88.67%
−/−/+/+	92.41%
−/+/−/+	95.233%

Notes: The driving potential amplitude is *V_D_* = 14 V, while the gate voltage is *V_G_* = 11.375 V for positive gate polarity “+” and *V_G_* = 2.625 V for its negative counterpart “−”.
